# Advancements in the investigation of gut microbiota-based strategies for stroke prevention and treatment

**DOI:** 10.3389/fimmu.2025.1533343

**Published:** 2025-03-04

**Authors:** Min Wang, Yan Liu, Li Zhong, Fang Wu, Jinjin Wang

**Affiliations:** Department of Gastroenterology, The First People’s Hospital of Xiaoshan District, Hangzhou, Zhejiang, China

**Keywords:** stroke, MGBA, neurotransmitter, immunity, SCFAs, TMAO, probiotics, FMT

## Abstract

Stroke represents a predominant cause of mortality and disability on a global scale, impacting millions annually and exerting a considerable strain on healthcare systems. The incidence of stroke exhibits regional variability, with ischemic stroke accounting for the majority of occurrences. Post-stroke complications, such as cognitive impairment, motor dysfunction, and recurrent stroke, profoundly affect patients’ quality of life. Recent advancements have elucidated the microbiota-gut-brain axis (MGBA), underscoring the complex interplay between gut health and brain function. Dysbiosis, characterized by an imbalance in gut microbiota, is significantly linked to an elevated risk of stroke and unfavorable outcomes. The MGBA plays a crucial role in modulating immune function, neurotransmitter levels, and metabolic byproducts, which may intensify neuroinflammation and impair cerebral health. This review elucidates the role of MGBA in stroke pathophysiology and explores potential gut-targeted therapeutic strategies to reduce stroke risk and promote recovery, including probiotics, prebiotics, pharmacological interventions, and dietary modifications. However, the current prevention and treatment strategies based on intestinal flora still face many problems, such as the large difference of individual intestinal flora, the stability of efficacy, and the long-term safety need to be considered. Further research needs to be strengthened to promote its better application in clinical practice.

## 1 Introduction

The global prevalence of stroke is on the rise (1, 2). Annually, the worldwide incidence of stroke is estimated to be between 15 and 20 million cases. The incidence rate varies across different countries and regions, typically ranging from 120 to 180 per 100,000 individuals (3). Ischemic stroke is the most common type, comprising approximately 60% to 70% of all stroke cases globally (4, 5). In contrast, hemorrhagic stroke, which includes cerebral hemorrhage and subarachnoid hemorrhage, has a lower incidence rate, accounting for about 30% to 40% of all strokes (6, 7). Following the onset of a stroke, a sequence of cascading reactions ensues, encompassing disturbances in energy metabolism, inflammation, immune responses, and cellular apoptosis (8, 9). Additionally, it may lead to various complications such as pulmonary infections, pressure ulcers, deep vein thrombosis, dysphagia, cognitive and psychological disorders, disuse syndrome, and epilepsy, among others (10–12). Numerous patients frequently experience unfavorable prognoses, resulting in elevated mortality and morbidity rates. Survivors may suffer from limb paralysis, speech disorders, and various other disabilities. Furthermore, the significant risk of recurrent stroke imposes considerable economic and psychological burdens on both families and society (13, 14).

In recent years, the discovery and comprehensive investigation of the MGBA has led to an increased understanding of the intricate relationship between gut microbiota and stroke (15, 16). On the one hand, stroke can significantly influence gut microbiota, as patients often exhibit reduced gastrointestinal motility, dietary modifications, and immune dysregulation post-stroke. These changes can result in alterations in the composition and functionality of the gut microbiota, including diminished microbial diversity, a decline in beneficial bacterial populations, and an increase in pathogenic bacteria (17, 18). On the other hand, the gut microbiota plays a significant role in the incidence and progression of stroke. Alterations in the gut microbiota can potentially intensify cerebral inflammation, impede neural repair mechanisms, and exacerbate brain injury by impairing the endothelial function of blood vessels (19, 20). Researchers are investigating the influence of the gut microbiota and its metabolic byproducts on stroke pathogenesis, particularly through their effects on the host’s immune response, inflammation levels, and neural transmission (21). Certain beneficial bacteria within the gut microbiota, including genera such as *Bifidobacterium, Lactobacillus, Enterobacter*, and *Lachnospira*, are postulated to confer protective effects against stroke. Conversely, specific gram-negative bacteria, such as *Clostridium* and *Escherichia coli*, can produce endotoxins or neurotoxins, which can induce systemic inflammation and disrupt nervous system function, consequently elevating the risk of stroke (22, 23). Metabolites produced by the gut microbiota, such as short-chain fatty acids (SCFAs), bile acids (BAs), tryptophan (Trp) metabolites, andtrimethylamine N-oxide (TMAO) (24, 25), are implicated in modulating brain health through their roles in neurotransmitter synthesis and metabolism, suppression of neuroinflammation, and stimulation of neurogenesis. Specifically, SCFAs have been shown to enhance cognitive function and regulate neurotransmitter activity (26); Trp metabolites are involved in mood and cognitive regulation and possess anti-inflammatory properties (27); BAs contribute to neurotransmitter regulation, inflammation inhibition, and cognitive enhancement (28); TMAO may increase the risk of stroke, exacerbate neuroinflammation, and affect cognitive function (29). Moreover, the gut microbiota may affect the incidence of strokes by modulating the integrity of the intestinal barrier function. A compromised intestinal barrier allows harmful substances, such as bacterial endotoxins, to translocate into the bloodstream, thereby activating the immune system and eliciting inflammatory responses that can exacerbate the pathophysiological processes associated with stroke (30, 31). Consequently, modulating the composition of the gut microbiota through the adjustment of intestinal immune and metabolic functions may facilitate the restoration of equilibrium within the intestinal microenvironment (16, 32), thereby offering a novel strategy for the comprehensive management of stroke. The modulation of gut microbiota through dietary adjustments (33), supplementation with probiotics and prebiotics (34, 35), pharmacological interventions (36), and fecal microbial transplantation (FMT) (37) aims to reduce stroke risk and enhance patient prognosis. These approaches offer novel strategies for stroke prevention and treatment. However, further clinical validation is required to ascertain their efficacy and safety (31).

## 2 MGBA

In recent years, there has been an increasing volume of research examining the interaction between the MGBA and stroke. This extensive investigation not only deepens our understanding of the pathogenesis of stroke but also offers novel insights into its prevention and treatment.

### 2.1 Definition and composition

The MGBA represents a multifaceted and significant physiological concept essential for maintaining human health (38). This axis denotes a bidirectional communication network linking the gut microbiota, the gastrointestinal tract, and the brain, mediated through neural, endocrine, and immune pathways (39). The gut microbiota consists of a diverse array of microorganisms, including bacteria, fungi, and viruses, which collectively establish a complex ecosystem within the gastrointestinal tract. This ecosystem exerts a profound influence on human digestion, metabolism, and immune functions (40). The gastrointestinal tract serves as a crucial organ linking the microbiota and the brain, playing a vital role not only in the digestion and absorption of nutrients but also in facilitating communication with the brain through the enteric nervous system, the endocrine system, and the immune system (41, 42). Conversely, the brain exerts regulatory control over the gut via these same systems, simultaneously responding to gut-derived signals that influence mood, cognition, and behavior (43–45).

### 2.2 Communication pathways

**Figure 1:**
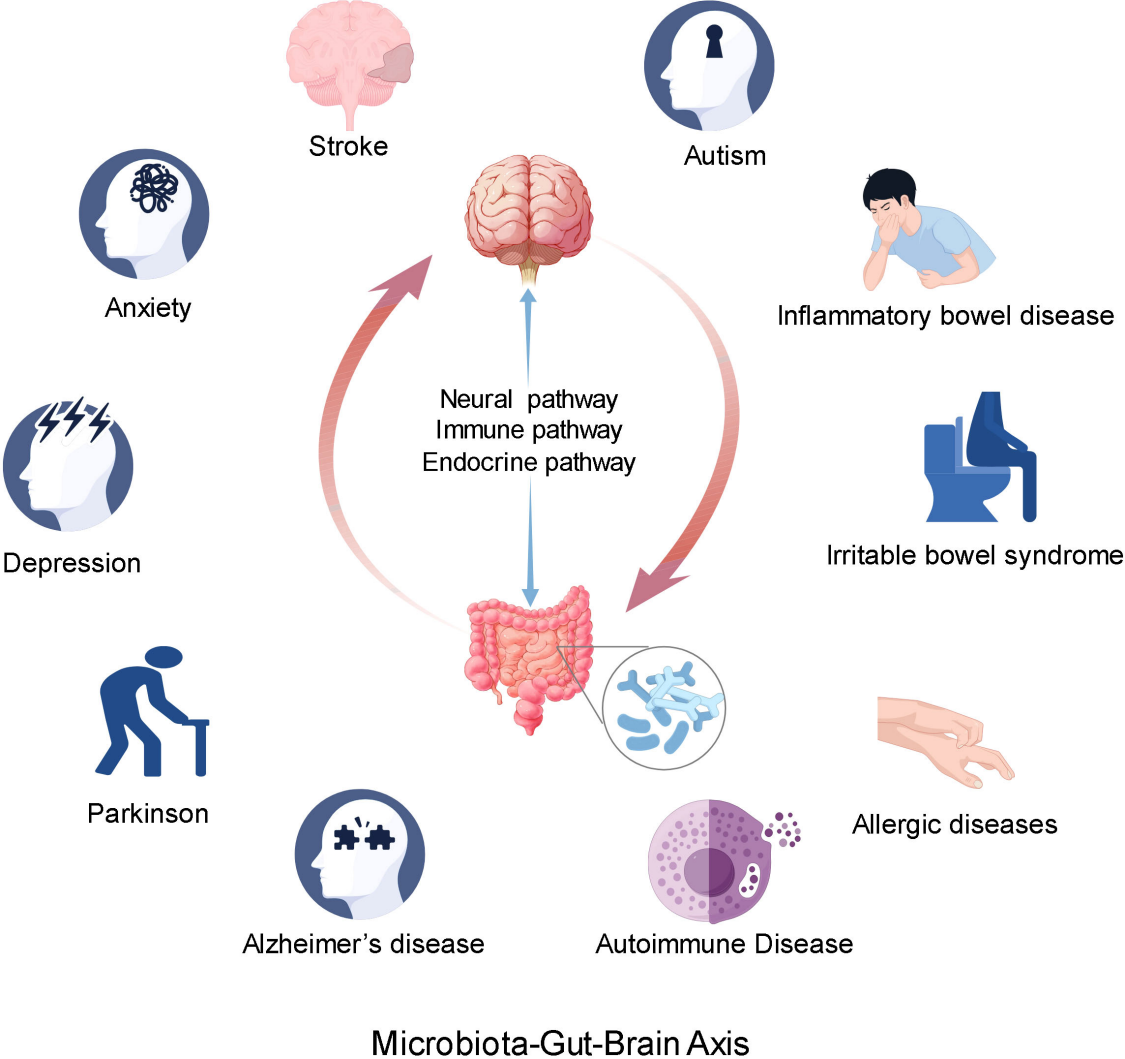
The microbial-gut-brain axis communicates primarily through neural, endocrine, and immune pathways. The abnormality of gut microbiota may be related to the occurrence and development of nervous system diseases and some immune system diseases. At the same time, the abnormal function of the brain may also affect the function of the digestive system.

The MGBA primarily facilitates communication via neural, endocrine, and immune pathways (46, 47) (**Figure 1**). Gut microbes produce metabolites and neurotransmitters, among others. On the one hand, it can stimulate the enteric nerve, which regulates intestinal function through local reflexes and communicates with the vagus nerve. On the other hand, direct or indirect activation of the vagus afferent fibers, the brain receives signals from the vagus efferent fibers to regulate the gut and microbes (48, 49). The metabolites generated by the gut microbiota influence the endocrine cells within the gastrointestinal tract, prompting the secretion of hormones, including serotonin (5-HT) and various intestinal peptides. These hormones subsequently enter the circulatory system and interact with neural receptors in the brain, thereby modulating neural activity (50, 51). Furthermore, the hypothalamus-pituitary-adrenal (HPA) axis is involved in the regulation of gastric and intestinal functions. Neurons located in the paraventricular nucleus of the hypothalamus release corticotropin-releasing hormone (CRH), which subsequently stimulates the anterior pituitary gland to produce adrenocorticotropic hormone (ACTH). Once in the bloodstream, ACTH prompts the adrenal cortex to synthesize and release glucocorticoids, such as cortisol. These glucocorticoids are integral to a variety of physiological processes, including the regulation of metabolism, the suppression of immune responses, and the affection of nervous system function (52, 53). The intestinal mucosa is densely populated with various immune cells, including macrophages, dendritic cells, and lymphocytes. Microbial components, such as peptidoglycan found in bacterial cell walls, are detectable by pattern recognition receptors, such as Toll-like receptors. Upon detection, these immune cells become activated and subsequently secrete cytokines and chemokines (54, 55). These molecules possess the capacity to modulate local immune responses within the intestine and augment the function of the intestinal barrier. Conversely, they are also capable of transmitting signals to the brain via the circulatory system or neural pathways. Upon receiving these signals, the brain orchestrates a regulatory response through the HPA axis, among other pathways (52).

### 2.3 Effect on health

Alterations in the gut microbiota may be associated with the onset and progression of various neurological disorders, including stroke, depression, anxiety, autism, Parkinson’s disease, and Alzheimer’s disease (56, 57). Such changes in the gut microbiota can influence the enteric nervous system, central nervous system, and immune system, potentially compromising barrier function (58, 59). Concurrently, the gut microbiota can modulate brain function by impacting the synthesis, metabolism, and signal transmission of neurotransmitters (60, 61). For instance, the gut microbiota has the capacity to synthesize serotonin, a neurotransmitter that can influence mood, cognition, and behavior (62, 63). Furthermore, an imbalance in the gut microbiota, known as dysbiosis, may contribute to the development of gastrointestinal disorders such as inflammatory bowel disease and irritable bowel syndrome (64, 65). These disorders have the potential to impact brain function via the MGBA, thereby inducing alterations in mood, cognition, and behavior (43). For instance, Cheng et al. have provided pioneering evidence that irritable bowel syndrome (IBS) is associated with neurological health issues, encompassing anxiety, depression, and cognitive deficits, as evidenced through neuroimaging, behavioral assessments, biochemical analyses, and genetic investigations (66). Furthermore, aberrations in cerebral function may influence gastrointestinal operations, exemplified by stress-induced gastric and intestinal dysfunction (67). Moreover, an imbalance in gut microbiota may contribute to immune-related disorders, including autoimmune and allergic diseases (68, 69) (**Figure 1**).

In summary, the MGBA constitutes a sophisticated and vital physiological system integral to human health. Achieving a thorough understanding of the mechanisms and functions of the MGBA holds the potential to provide significant insights into the development of innovative therapeutic interventions and pharmaceuticals, while also offering new perspectives and strategies for improving overall health.

## 3 The impact of gut microbiota on the pathophysiological process of stroke

The MGBA constitutes a bidirectional communication network linking the gastrointestinal tract and the brain, encompassing neural, endocrine, and immune pathways. Through the MGBA, the gut microbiota can influence brain function and behavior (70).

### 3.1 Neural regulation

#### 3.1.1 Vagus nerve regulation

**Figure 2:**
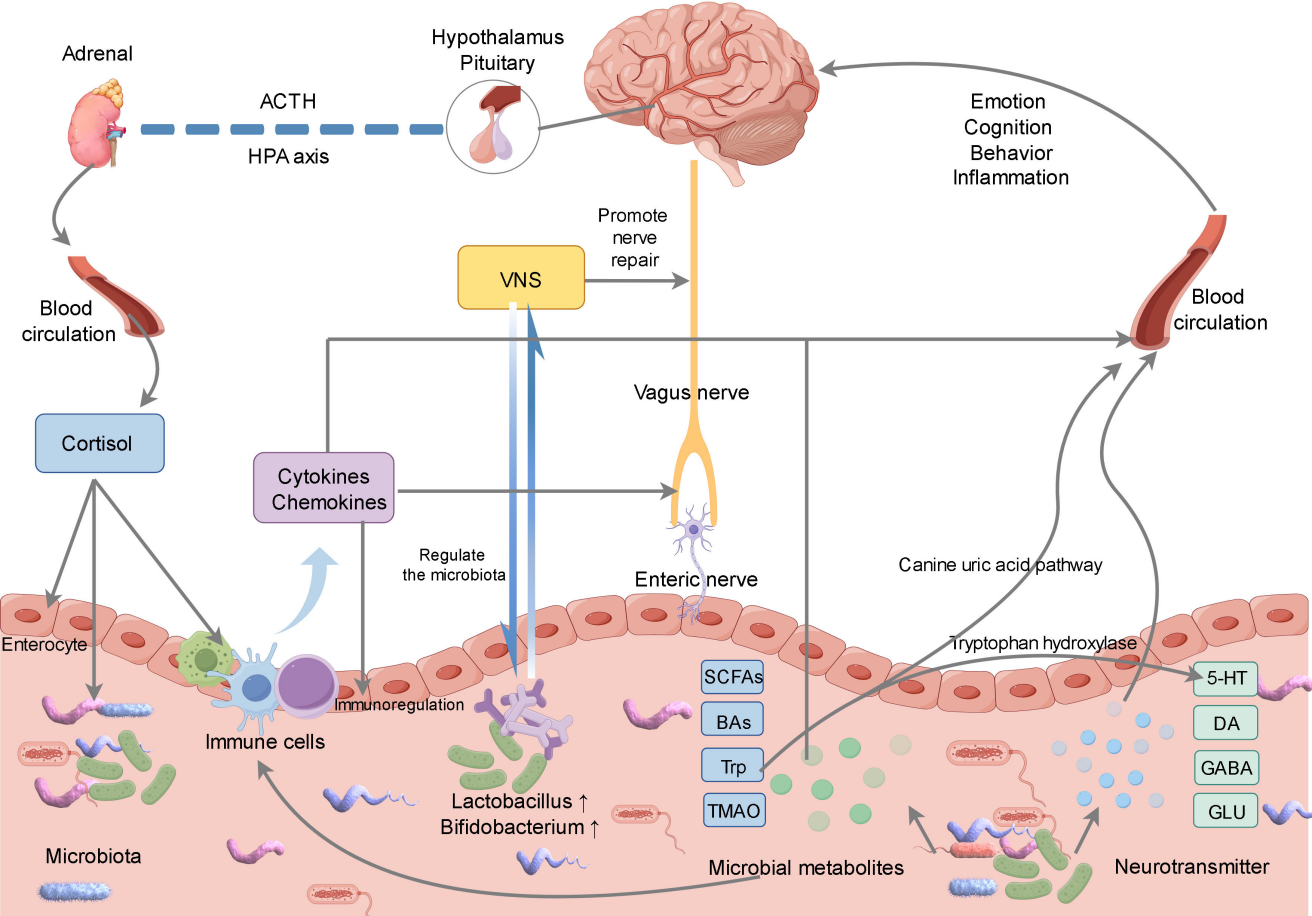
Gut microbiota can affect the brain through neural, endocrine, immune and other pathways. Gut microbiota can stimulate gut neurons, which transmit signals to the brain through the vagus nerve. Meanwhile, vagus nerve stimulation can not only promote nerve repair, but also promote the regulation of gut microbiota. In addition, we can affect brain health by regulating the metabolites of gut microbiota and the synthesis and metabolism of neurotransmitters, and stimulate the immune system to release cytokines to affect the functions of the gut and brain through immune pathways. The brain affects the physiological function of the gut through the hypothalamic-pituitary-adrenal axis, and indirectly regulates the microbial community.

The vagus nerve plays a crucial role in the MGBA, facilitating bidirectional communication between the enteric nervous system (ENS) and the brain (60, 71, 72). The ENS, an autonomous nervous system embedded within the gastrointestinal wall, comprises a substantial network of neurons and glial cells. It is responsible for regulating intestinal motility, secretion, and immune functions (73, 74). Simultaneously, the ENS is capable of detecting a range of stimuli within the gastrointestinal tract, including chemical substances, mechanical stimuli, and temperature variations, subsequently transducing this information into neural signals (38, 73, 75) (**Figure 2**). Furthermore, the gut microbiota can modulate brain activity and emotional states by stimulating the ENS and the vagus nerve (76, 77).

Following a stroke, alterations in the gut microbiota may activate nerve endings within the intestinal wall, thereby transmitting signals to the brain via the vagus nerve (78). Concurrently, vagus nerve stimulation (VNS) exerts beneficial effects through multiple mechanisms. For instance, VNS modulates the gut microbiota, attenuates inflammatory responses, regulates the permeability of the blood-brain barrier, promotes angiogenesis, and facilitates axon regeneration, among other effects (79–82) (**Figure 2**). Jiang et al. demonstrated that VNS effectively modulates mast cell degranulation, mitigates damage to both the blood-brain barrier and colonic barrier post-stroke, ameliorates dysbiosis of the rat gut microbiota, and attenuates inflammatory responses (83). Similarly, Laureys et al. reported in their rat studies that VNS influences the regulation of microglial and astrocytic activity, enhances the oligodendrocytes’ clearance capacity following the initial injury, and significantly facilitates myelination and synaptic regeneration (84). Park et al. demonstrated that VNS-regulated MGBA altered intestinal morphology and the composition of gut microbiota, notably increasing the abundance of *Bifidobacterium*, which facilitated neuroprotection following a stroke (85). Araujo et al. also found that the Brunner’s gland in the duodenum could mediate the enrichment of Lactobacillus in the intestine after VNS, promoting neural recovery (86). Currently, numerous clinical trials suggest that the integration of VNS with rehabilitation training positively influences the enhancement of motor function recovery in stroke patients. Following the administration of VNS in conjunction with synchronized rehabilitation training, there is a marked improvement in the patient’s limb motor function (79, 87). Investigating the mechanisms through which VNS modulates gut microbiota and enhances stroke prognosis is anticipated to offer novel strategies for stroke treatment.

#### 3.1.2 Neurotransmitter regulation

The gut microbiota is capable of producing a variety of neurotransmitters and neuroregulatory compounds, such as 5-hydroxytryptamine (5-HT), dopamine (DA), gamma-aminobutyric acid (GABA), and glutamic acid (GLU), among others (88, 89) (**Figure 2**). These compounds can traverse the bloodstream to reach the brain, where they influence neural transmission and brain function (90, 91). Following a stroke, changes in the gut microbiota may affect the balance of neurotransmitters, modulate the excitatory and inhibitory functions of the nervous system, and facilitate the recovery of neural functionality (15, 16). FMT from stroke animals can also affect the neurobehavior, neurotransmitter levels and other aspects of recipient animals by changing the gut microbiota environment (92, 93).

Trp is the only precursor of 5-HT, and the dysregulation of Trp metabolite products plays a central role in the pathogenesis of many neurological and psychiatric disorders (94). In mouse experiments, Gao et al. discovered that enhancing Trp hydroxylase expression and boosting 5-HT levels in the brain and colon led to enhanced synapse formation and astrocyte maintenance (95). Branco et al. found in mice exposed to endotoxin that injection of dibutyl phthalate (DIZE) could promote intestinal microbiota regulation and increase central 5-HT levels, activate the efferent sympathetic nerve arm of the inflammatory reflex, and alleviate the inflammatory response in mice (96). Furthermore, GABA can enhance intestinal barrier function by regulating intestinal mucin and tight junction proteins (97). Sukhraj Kaur et al. found that L18, a promising GABA-secreting bacterium, could increase the concentration of connexin proteins and regulate the intestinal microbiota (98). In another animal experiment, Wu et al. found that Lactobacillus plantarum L5, a high GABA-producing strain, reshaped the intestinal microbiota composition and increased GABA levels in mice, thereby alleviating central nervous system inflammation (99). Nonetheless, this field continues to face certain limitations, including ambiguous mechanisms of action and the absence of standardized clinical protocols. Ongoing research endeavors to elucidate the interactions between the gut microbiota and neurotransmitters in the post-stroke condition, potentially yielding novel insights and strategies for the development of enhanced stroke treatments.

### 3.2 Metabolic regulation

The gut microbiota plays a significant role in the host’s metabolic processes, influencing the digestion, absorption, and metabolism of various nutrients (100, 101) (**Figure 2**).

#### 3.2.1 SCFAs metabolism regulation

The gut microbiota significantly influences the synthesis and degradation of fatty acids. It possesses the capability to decompose dietary fiber via fermentation processes, leading to the generation of SCFAs such as acetic acid, propionic acid, and butyric acid (102). SCFAs play a multifaceted role in physiological processes, including the regulation of epithelial barrier function and the immune system. They contribute to the maintenance of intestinal mucosal integrity and possess the ability to traverse the blood-brain barrier (BBB), thereby influencing neurotransmitter synthesis and release (91). Additionally, SCFAs are involved in the regulation of nervous system function and provide an energy source for neurons, which enhances neuronal survival and facilitates functional recovery (103). Furthermore, SCFAs exhibit neuroprotective properties and modulate inflammatory cytokines by inhibiting the activation of microglia and astrocytes (104). They also influence the regulation of the occludin protein by stimulating microglia, thereby impacting the integrity of the BBB (104). Ning et al. identified a significant enrichment of *Escherichia coli, Ruminococcaceae, Enterobacter cloacae, Streptococcus*, and *Lactobacillus* in the intestinal microbiota of rats with cerebral infarction. This enrichment of opportunistic pathogens was frequently associated with a poor prognosis. Conversely, an increased presence of SCFAs-producing bacteria, including *Fusicatenibacter, Ruminococcaceae, Eisenbergiella*, and *Faecalibacterium*, was often correlated with more favorable prognostic outcomes (105). Gu et al. demonstrated that the intestinal microbiota has the potential to inhibit hippocampal neuron apoptosis in rats experiencing cerebral ischemia by elevating systemic levels of SCFAs. Furthermore, prolonged supplementation with SCFAs was shown to mitigate the inflammatory response and enhance neuroprotective effects following cerebral ischemia (106). Furthermore, Zhao et al. demonstrated that the intervention in cerebral ischemia through the transplantation of SCFAs-rich bacteria and the supplementation of butyrate constitutes an effective approach by modulating the intestinal microbiota (107). Butyrate is highly valued for its ability to maintain the integrity of the intestinal barrier function, thereby promoting intestinal health and epithelial integrity. This is achieved through the stimulation of tight junction protein expression and the production of mucin by goblet cells (108). Moreover, butyrate demonstrates significant potential in lowering blood lipid levels, modulating hemorheology, inhibiting histone deacetylase activity, reducing inflammation, promoting angiogenesis, and maintaining the integrity of the BBB (109–111). To summarize, the gut microbiota and SCFAs contribute to neurological recovery following a stroke through a variety of mechanisms.

#### 3.2.2 Regulation of BAs metabolism

After a stroke, the regulation of intestinal microbiota and BAs plays a crucial role in facilitating neural recovery. The gut microbiota and BAs have a mutual relationship, where the gut microbiota processes BAs and controls their makeup, while BAs can impact the structure and operation of the microbial community. Both have crucial functions in the digestive system and metabolic disorders (112–114). The metabolic capabilities of intestinal bacteria with respect to BAs vary, leading to alterations in the BAs spectrum composition as the intestinal microbiota changes (115). Certain BAs compositions may positively influence neural recovery following a stroke (116). For example, some BAs metabolites may possess anti-inflammatory, antioxidant, or neuroprotective properties (117). Additionally, BAs are crucial in the digestion and absorption of fats and in the regulation of glucose, steroid levels, and energy homeostasis (118–120). Collectively, these functions contribute to neurological recovery.

BAs are capable of activating a range of receptors, including the farnesoid X receptor (FXR) and the G protein-coupled BAs receptor 1 (TGR5). These receptors are extensively expressed within the nervous system and play a crucial role in modulating neuronal function and survival (121). Activation of FXR and TGR5 influences neuroinflammatory responses, neurogenesis, and neuroplasticity, thereby facilitating neural recovery following stroke (122). Gao et al. found that in hyperlipidemic mice, BAs synthesis increased concomitantly with elevated levels of fecal alkaline phosphatase and Ruminococcaceae UCG-010, and was facilitated via the intestinal FXR-fibroblast growth factor 19 (FXR-FGF19) axis, thereby enhancing lipid absorption (123). In a separate study, He et al. reported a decrease in intestinal microbiota-mediated BAs, particularly ursodeoxycholic acid (UDCA), in patients with brain stroke. In murine models, the restoration of UDCA was achieved by inhibiting NLRP3-related pro-inflammatory cytokines through the TGR5/PKA signaling pathway, which reduced the mouse infarction area, and improved neurological function and cognitive function (124). The regulation of BAs by the gut microbiota is mediated through various mechanisms, which interact with each other to promote neural recovery after stroke.

#### 3.2.3 Trp metabolic regulation

Initially, Trp can be metabolized into 5-hydroxyTrp (5-HTP) within the central nervous system and enteric chromaffin cells, subsequently leading to the production of 5-HT (125). Serotonin plays a crucial role in modulating adaptive responses and reactions to environmental changes, and is significantly involved in mood regulation, sleep modulation, cognitive function, and gastrointestinal motility (126). In animal models of cerebral infarction, 5-HT receptors have been identified as promising targets for neuroprotective strategies (127). Agonists targeting the 5-HT1A receptor have shown potential in preventing neuronal damage resulting from transient focal or global cerebral ischemia (128). Secondly, the majority of Trp is metabolized via the canine uric acid pathway. In this process, Trp is initially converted into N-formylkynurenine through the action of either indoleamine 2,3-dioxygenase (IDO) or Trp 2,3-dioxygenase (TDO). Subsequently, it undergoes a series of enzymatic reactions to produce canine uric acid, 3-hydroxycanine uric acid, and 3-hydroxyorthoaminobenzoic acid (129). The metabolic products of this pathway possess functions related to immune regulation, antioxidation, neurotransmission, neuroprotection, and modulation of neural plasticity, all of which play significant roles in the pathogenesis of neuroinflammatory diseases (130–132). Moreover, intestinal microorganisms possess the capability to directly metabolize Trp into indole and its derivatives. These compounds can modulate the expression of both pro-inflammatory and anti-inflammatory cytokines, thereby contributing to the maintenance of intestinal homeostasis. The preservation of intestinal homeostasis is crucial for mitigating inflammatory responses and safeguarding the function of the intestinal mucosal barrier (133, 134). Improving the functionality of the intestinal mucosal barrier can reduce the translocation of harmful substances, such as endotoxins, into the circulatory system. This reduction subsequently diminishes systemic inflammatory responses and indirectly supports recovery post-stroke. Shin’s research, utilizing clinical trials and metabolomics analysis, demonstrated that indole-3-propionic acid (IPA), synthesized by gut microbiota, exerts a protective effect on microglia against inflammatory damage. This protective role enhances neuronal function, thereby establishing IPA as a crucial mediator in the gut-brain axis interaction (135). Giovanni et al. also found that the intestinal microbiota and the serum metabolite IPA derived from intestinal bacteria can promote axon regeneration and functional recovery through immune-mediated mechanisms (136).

#### 3.2.4 Regulation of metabolism of TMAO

TMAO primarily originates from choline, phosphatidylcholine, and L-carnitine found in food. The intestinal microbiota metabolizes these compounds into trimethylamine (TMA), which is subsequently absorbed into the liver and oxidized by flavin monooxygenase (FMO) to form TMAO (137–139). Elevated levels of TMAO are regarded as a risk factor for cardiovascular disease, which in turn is a significant risk factor for stroke (140, 141). TMAO may contribute to an increased risk of stroke by promoting atherosclerosis, enhancing platelet activity, and elevating the risk of thrombosis (142–144). TMAO is closely related to the risk of stroke, the severity of the stroke, and the prognosis (145, 146).

Following a stroke, both the inflammatory response and oxidative stress are pivotal in contributing to neurological damage and dysfunction (29). TMAO can penetrate the central nervous system, thereby inducing neuroinflammation and immune responses, compromising the integrity of the BBB, and elevating the expression of amyloid-beta (Aβ) and hyperphosphorylated tau. This process involves the regulation of several signaling pathways, including NLRP3/ASC/caspase-1, PERK/eIF2α/ER-stress, SIRT3/SOD2-mtROS, SIRT1/p53/p21/Rb, TXNIP-NLRP3, and PERK/Akt/mTOR. These pathways collectively stimulate inflammation, apoptosis, endoplasmic reticulum stress, and the production of reactive oxygen species (ROS) (147). Sun et al. identified that TMAO facilitates the activation of the NLRP3 inflammasome in microglia via the FTO/IGF2BP2 pathway, thereby exacerbating neurological damage resulting from ischemic stroke (148). Furthermore, TMAO initiates pro-inflammatory pathways, such as NF-κB signaling, leading to the activation of inflammatory cells and an increase in the secretion of inflammatory mediators. These processes collectively enhance inflammation and oxidative stress, further impair neurological function, and elevate the risk of stroke recurrence (29, 149). Moreover, TMAO plays a role in modulating the expression of various microRNAs (miRNAs) that are involved in neuroinflammation, oxidative stress, and neuronal apoptosis. The gut microbiota-miRNA-brain axis has emerged as a novel mechanism linking gut dysbiosis to neurological disorders (150). The regulation of TMAO levels by the gut microbiota may confer neuroprotection by mitigating inflammation and oxidative stress, thereby facilitating neurological recovery post-stroke (151, 152). Consequently, maintaining optimal TMAO levels and a balanced gut microbiota may represent a promising therapeutic strategy for the treatment and recovery of stroke complications.

### 3.3 Immune regulation

Gut microbiota and immune regulation are closely related and jointly affect stroke. Microbiota plays a role in the pathogenesis of stroke through metabolites and balance, and immune regulation imbalance affects inflammation and immune cells. They interact with each other in the whole process of stroke, and in-depth exploration of their relationship is conducive to the prevention and treatment of stroke (153) (**Figure 2**).

#### 3.3.1 Regulate intestinal immunity and maintain intestinal barrier integrity

The intestinal microbiota engages in interactions with both innate immune cells, including macrophages and dendritic cells, and adaptive immune cells, such as T cells and B cells, within the intestinal environment. These interactions play a crucial role in modulating the activity and function of these immune cells, thereby maintaining a balanced state of intestinal immunity (154, 155). Mahajan et al. provided evidence through murine studies that *Lactobacillus strains 3630* and *3632* possess the capability to sustain intestinal immune homeostasis (156). In addition, the transcription factor GATA4 is related to and interacts with intestinal flora. Intestinal flora can affect the expression and function of GATA4 through metabolites, immune regulation, intestinal barrier maintenance and other ways, and GATA4 is also closely related to intestinal flora in the regulation of intestinal related physiological processes. Natalia Shulzhenko et al. found in mouse experiments that transcription factor GATA4 controlled intestinal bacterial colonization and inflammatory tissue immunity by regulating retinol metabolism and luminal IgA. In mice without GATA4 expression, commensal segmental filamentous bacteria promote a pathogenic inflammatory immune response that disrupts barrier function (157). Sandra Morais Cardoso et al. further revealed that the microbial toxin β-n-methylamino-L-alanine (BMAA) can lead to the depletion of segmented filamentous bacteria (SFB), which play a crucial role in regulating intestinal immunity. This depletion results in dysbiosis, the migration of immune cells, heightened intestinal inflammation, and impaired barrier integrity. Furthermore, BMAA has been shown to induce mitochondrial dysfunction, thereby activating neuronal innate immunity (158).

Additionally, various immune cell types, including regulatory T cells (Treg), regulatory B cells (Breg), and innate lymphoid cells (ILCs), as well as immune-suppressive cells like tolerogenic macrophages (tMacs), tolerogenic dendritic cells (tDCs), myeloid-derived suppressor cells (MDSCs), and inflammatory cells such as inflammatory macrophages (iMacs), CD4+ T helper cells (Th1, Th2, Th17), natural killer T cells (NKT), and neutrophils, are capable of expressing diverse receptors for microbial metabolites, including SCFAs, BAs, and TMAO (159, 160). The activation of these receptors not only promotes the differentiation and function of immune-suppressive cells but also inhibits inflammatory cells, leading to the reprogramming of the local and systemic immune system to maintain the individual’s internal environment balance (54, 160). For example, SCFAs influence the function of the intestinal barrier and systemic immunity through direct interactions with intestinal epithelial cells, phagocytes, B cells, plasma cells, and regulatory Tregs (161). Concurrently, BAs perform their immunomodulatory functions by binding to BAs receptors (BARRs) present on monocytes, tissue-resident macrophages, Th17 cells, Tregs, dendritic cells, and NKT cells (162).

#### 3.3.2 Reduce neuroinflammation

After a stroke, a neuroinflammatory response is triggered, leading to neuronal damage and neurological dysfunction. The gut microbiota plays a crucial role in modulating the immune system by inhibiting the activation of inflammatory cells and reducing the secretion of pro-inflammatory mediators, such as tumor necrosis factor-α and interleukin-1β, thereby mitigating neuroinflammation (163, 164). Additionally, specific gut microbiota can promote the proliferation of regulatory T cells and suppress excessive immune responses, thereby safeguarding the nervous system (32). Wang et al. demonstrated that Th17 cells, specific to the intestinal environment, possess immune regulatory functions capable of inhibiting effector T cell activity both *in vitro* and *in vivo*, mediated by interleukin-10 (IL-10) and the transcription factor c-Maf (165). Additionally, microglia, as the innate immune cells of the central nervous system, become activated post-stroke, subsequently releasing inflammatory mediators that exacerbate neuronal damage (166). Lu et al. found that tofacitinib (TOF) can regulate the activation of neuroinflammatory microglia after neurological injury through the JAK/STAT pathway, which has important implications for immunotherapy of neurological injury (167). Jeffrey L et al. demonstrated that the equilibrium between neurotoxic and neuroprotective astrocytes is modulated by a specific pool of cyclic adenosine monophosphate (cAMP) originating from soluble adenylate cyclase. Furthermore, they found that the proliferation of neuroprotective astrocytes suppresses microglial activation and the subsequent differentiation into neurotoxic astrocytes, thereby enhancing neuronal survival (168).

In summary, MGBA promotes recovery after stroke through various mechanisms, including neural regulation, metabolic regulation, and immune regulation. By further studying these mechanisms, it is hoped that new strategies and methods for the prevention and treatment of stroke can be provided.

## 4 Therapeutic strategies based on gut microbiota

**Figure 3:**
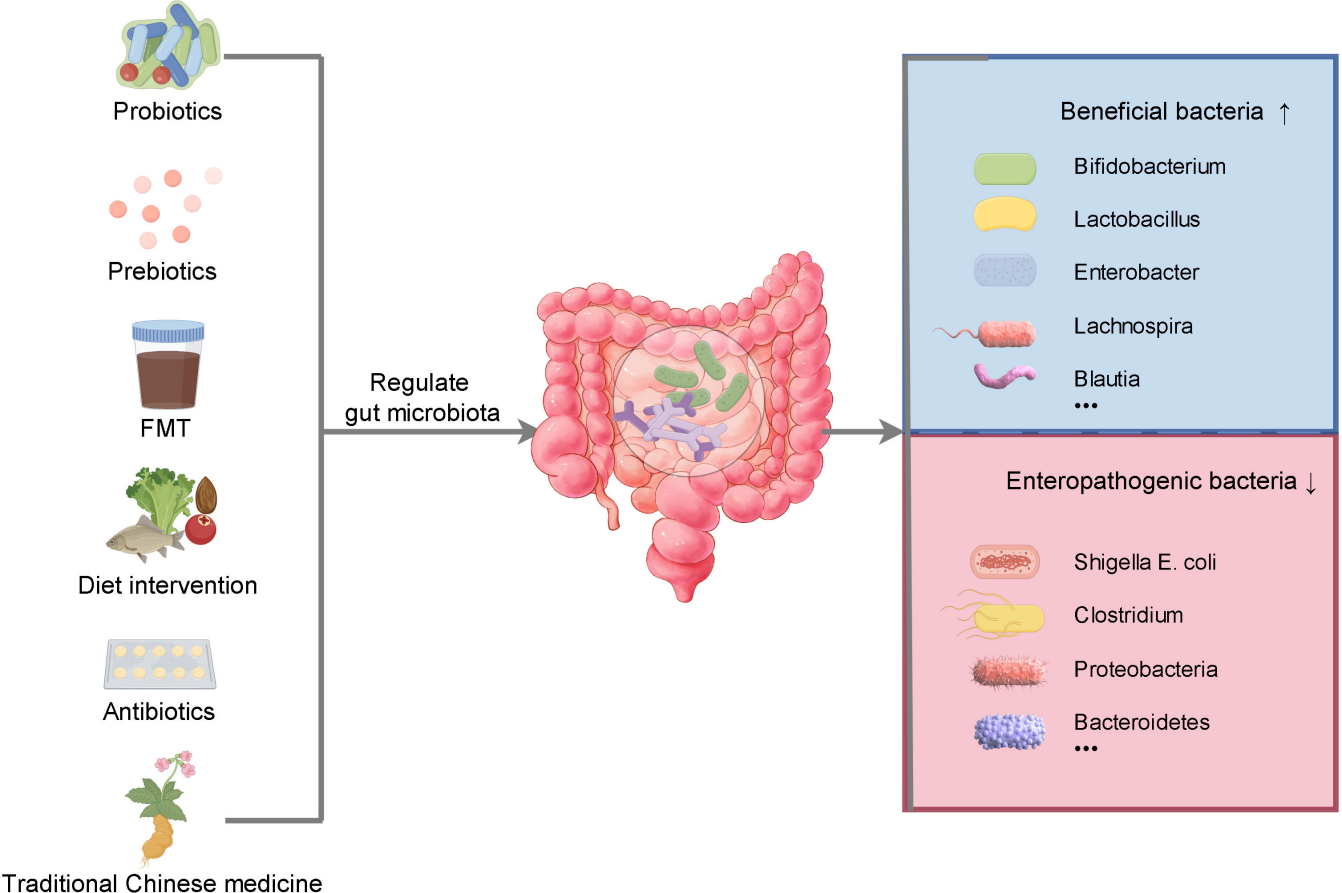
The regulation of gut microbiota can be achieved in many ways. Supplementation of probiotics and prebiotics, fecal microbiota transplantation, dietary intervention, rational use of antibiotics and traditional Chinese medicine can increase the abundance of beneficial bacteria, reduce the abundance of opportunistic bacteria, maintain the balance of intestinal flora and promote health.

Emerging targeted therapeutic strategies centered on the modulation of gut microbiota present promising avenues for the prevention and treatment of stroke. These strategies encompass the administration of probiotics to restore gut equilibrium, the use of prebiotics to promote the growth of beneficial bacteria, the implementation of FMT to re-establish a healthy microbiome, and dietary interventions such as the Mediterranean diet, which is recognized for its anti-inflammatory and neuroprotective properties. Probiotics, especially strains like *Bifidobacterium* and *Lactobacillus*, have been demonstrated to improve gut barrier integrity, mitigate systemic inflammation, and promote neural health. FMT offers a novel technique for restoring beneficial gut microbiota, whereas the consumption of prebiotics cultivates a conducive gut environment. When integrated with dietary modifications, these strategies have the potential to enhance stroke outcomes by optimizing gut health and minimizing risk factors (**Figure 3**).

In the investigation of therapeutic and preventive strategies for stroke, the significance of gut microbiota is gaining increasing recognition. This section seeks to elucidate treatment strategies by conducting an in-depth analysis of the interaction between gut microbiota and stroke, with a focus on modulating the balance of the gut microbiota.

### 4.1 Application of probiotics and prebiotics

Probiotics, as active microorganisms, play a crucial role in regulating the balance of gut microbiota, enhancing intestinal barrier function, inhibiting the proliferation of pathogenic bacteria, and mitigating inflammatory responses (56, 169). Numerous studies have indicated that specific probiotic strains, such as *Bifidobacterium* and *Lactobacillus*, can improve intestinal function, modulate immune responses, and optimize metabolic health in individuals recovering from stroke through various mechanisms (170–172). These findings underscore the potential therapeutic benefits of probiotics in the management and recovery of stroke. *Bifidobacterium* inhibits the growth of harmful bacteria to maintain the balance of intestinal flora, strengthen the intestinal barrier function, reduce the inflow of harmful substances into the blood, and reduce systemic inflammatory response to protect the nervous system (173). It is also involved in the metabolism of neurotransmitters and affects the neural activity of the brain through the transmission of signals by MGBA. In animal experiments and some human studies, it has shown potential neuroprotective value in the prevention of neurodegenerative diseases and delay of cognitive decline (174, 175). Lactobacillus colonize the intestinal mucosa, inhibit harmful bacteria, optimize the structure of bacterial flora, enhance the integrity of the intestinal barrier, reduce inflammatory factors into the blood and avoid brain neuroinflammation. It regulates the immune function of the body, inhibits excessive inflammation, communicates with the brain through MGBA, and plays a positive role in improving the emotional problems associated with intestinal tract, relieving neuroinflammation related to neurological diseases, and promoting the recovery of nerve function, reflecting the neuroprotective effect (176, 177). In animal models of stroke, probiotic supplementation can inhibit neuronal apoptosis and improve neurological dysfunction, showing anti-inflammatory and antioxidant properties (171). In a study by Deng et al., it was confirmed that the treatment effect of probiotics combined with enteral nutrition group was better than that of enteral nutrition alone group, and probiotics combined with enteral nutrition significantly reduced the occurrence of complications such as esophageal reflux, abdominal distension, constipation, diarrhea, gastric retention and gastrointestinal bleeding (178). In addition, the results of Huang et al. show that supplementing rats with Lactobacillus MH-022 can significantly improve motor function deficits, preserve dopaminergic neurons, enhance antioxidant capacity, and alleviate neuroinflammation by restoring mitochondrial function (179). In addition, Lu et al. improved the efficacy of *Lactiplantibacillus plantarum* (LP) in the intestine by using a layer-by-layer encapsulation technology, restoring the disordered intestinal microbial composition in mice, significantly increasing the level of SCFAs, and alleviating brain neuroinflammation and neuron damage in mice (180).

Prebiotics are non-digestible compounds that selectively enhance the growth and activity of beneficial intestinal microbiota, as they are not absorbed by the human body. Common examples of prebiotics include fructooligosaccharides, inulin, and galactooligosaccharides, among others (181). Prebiotics can modulate the composition of the gut microbiota by fostering the proliferation of beneficial bacterial species and enhancing the intestinal microecological environment. For instance, Zhao et al. found that Puerariae Lobatae Radix-resistant starch(PLR-RS) can improve intestinal flora imbalance, enrich *Akkermansia* and *Bifidobacterium*, and reduce brain damage and intestinal barrier dysfunction caused by ischemic stroke (182). Research conducted by Zhu et al. demonstrated that α-d-1, 3-glucan effectively augmented the abundance of intestinal bacterial taxa such as *Fltibacter*, *Butyricicoccus*, and *Oscillibacter*, as well as their metabolites, including lipopolysaccharides (LPS), BAs, and SCFAs. These changes resulted in improvements in inflammatory responses, lipid metabolism, and energy metabolism signaling pathways (183). Additionally, Li et al. demonstrated that inbred rats could be protected by inulin through the MGBA pathway in the medial ganglionic eminence neurons. Inulin significantly upregulated the mitogen-activated protein kinase signaling pathway in the hippocampus of rats and altered the composition of the gut microbiota, leading to an increase in the abundance of *Lactobacillus* and *Clostridium_sensu_stricto_1* in the gut of rats, while reducing the abundance of *Ruminococcus UCG_005, Prevotella_9, Oscillospiraceae*, and *Clostridia UCG_014* (184). In the future, with further research, it is expected that more precise and effective combinations of probiotics and prebiotics will be developed, providing new treatment strategies for stroke patients.

Both probiotics and prebiotics focus on the regulation of intestinal flora, and play a positive role in reducing the risk of stroke and promoting the rehabilitation of stroke patients by affecting the intestinal barrier function, metabolic function, and interacting with the central nervous system with the help of MGBA (34, 185). However, their specific effects are restricted by many factors, such as type, dose, duration of use, and the initial state of individual intestinal flora and overall health status. More in-depth research is still needed to clarify the best application method and exact effect in clinical practice (186). In conclusion, probiotics and prebiotics have shown potential positive effects on the prevention and rehabilitation of stroke. Reasonable intake of foods rich in them or the use of related supplements can be used as a beneficial means to assist the prevention and treatment of stroke, but should not replace regular medical treatment.

### 4.2 Fecal microbiota transplantation

FMT involves transferring the fecal microbiota of a healthy donor to the gut of a patient, which is expected to regulate the patient’s gut microbiota balance and subsequently affect the immune, nervous, and other physiological systems (37). Currently, FMT has shown good effects in treating some intestinal diseases such as Clostridium difficile infection and inflammatory bowel disease (187, 188). In stroke treatment, FMT also shows potential (32, 189). Some studies suggest that FMT may bring new hope for stroke patients’ recovery by regulating inflammation, promoting nerve regeneration, etc (92, 190). For example, FMT can improve inflammation by restoring microbial composition and function, and it can promote neuroprotection by lowering IL-17, IFN-γ, and Bax, and increasing Bcl-2 expression (189). Additionally, the gut microbiota and urate metabolism have significant protective effects on stroke, Zhang et al. further verified that blueberry extracts, through interactions with Prevotella, resulted in significant changes in urate levels, Trp, and indoleamine 2,3-dioxygenase levels, and played a neuroprotective role (191). Guo et al. proved that SCFAs mediated by MGBA improved brain stroke through DZSM. DZSM can significantly change the composition of the gut microbiota and significantly increase the production of SCFAs, thereby regulating the PI3K/AKT/caspase-3 pathway to inhibit neuronal cell apoptosis. FMT can reproduce the beneficial effects of DZSM on brain stroke and SCFAs (192).

However, research in this field is still in its early stage, and FMT has many points worth paying attention to for stroke patients. In safety, since stroke patients’ intestinal function may be affected by diseases, they are prone to intestinal flora imbalance after FMT, causing gastrointestinal discomfort such as diarrhea and abdominal distension. In addition, despite strict screening of donors, there is still a risk of transmission of unknown pathogens. At the same time, the body may also produce immune responses due to foreign bacteria, such as fever and rash. Threat to patient health (193). In terms of ethical considerations, fully informed consent of donors should be guaranteed, their privacy should be strictly protected, the principle, effects and risks of treatment should be fully informed to patients and their families, and their independent choice should be respected. Strict ethical review procedures should be followed especially for exploratory applications (194). In terms of clinical success rate, it is still in the exploratory stage in the treatment of stroke. Although some studies have shown that it has a certain positive effect on improving neurological function and promoting rehabilitation, it is affected by many factors such as the intestinal flora of the donor, the selection of transplantation method and timing, and individual differences (such as stroke type, basic health status, initial intestinal state, etc.). It is still difficult to define an exact success rate data, and the overall application needs to be carefully evaluated and weighed against the pros and cons (92).

### 4.3 Dietary intervention

Dietary intervention is making significant progress in promoting recovery from stroke.

First, the Mediterranean diet has received considerable attention. This dietary pattern is rich in fruits, vegetables, whole grains, legumes, nuts, and olive oil, with a moderate intake of fish and poultry, and a limited intake of red meat and sweets (195, 196). Research has indicated that the Mediterranean diet can lower the chances of experiencing a stroke and aid in the recovery of stroke survivors. The combination of its abundant antioxidants, unsaturated fatty acids, and dietary fiber has been shown to decrease inflammation, enhance vascular function, control lipid and glucose levels, and create a beneficial physiological environment for the recovery of neural function (197, 198).

Secondly, fermented foods are rich in probiotics, such as *Bifidobacterium* and *Lactobacillus acidophilus* in yogurt and lactic acid bacteria in pickles. These probiotics can regulate intestinal flora, inhibit the growth of harmful bacteria, enhance intestinal barrier function, reduce intestinal endotoxin and inflammatory factors into the blood circulation, and thus reduce the body’s inflammatory response (199, 200). At the same time, γ-aminobutyric acid and other components in fermented foods have a regulatory effect on the nervous system. When neuroinflammatory reaction occurs in the brain after stroke, it helps to reduce the damage of inflammation to nerve cells, improve the cognitive function and motor function of patients by affecting the metabolism of neurotransmitters and promoting the regeneration of nerve cells. Improve quality of life (201, 202). It also helps to improve the problems of slow intestinal peristalsis, weakened digestive function and constipation caused by limited activity and neurological function changes in stroke patients, and avoid the increase of abdominal pressure and blood pressure fluctuation caused by forced defecation, which is conducive to the stability and rehabilitation of the disease (199).

Dietary fiber also has many positive effects to cerebral apoplexy. It can reduce blood pressure by increasing the concentration of nitric oxide and reducing the absorption of sodium in the intestine. Reduce the absorption and synthesis of cholesterol and triglyceride to reduce blood lipids, reduce the level of inflammation, and reduce the chronic inflammatory reaction in the whole body including the brain (203); Fiber can provide substrates for the fermentation of intestinal flora and regulate the structure of intestinal flora, which is conducive to the production of SCFAs (204). Dietary fiber can combine with some substances such as choline in the intestine to reduce the formation of TMA and TMAO (205). In addition, dietary fiber can promote intestinal peristalsis, improve intestinal function, prevent constipation, and avoid blood pressure fluctuations caused by forced defecation (206). These effects can help to reduce the risk of stroke and promote the rehabilitation of stroke patients.

Polyphenols as a natural compounds exist widely in plant foods, have important influence on cerebral apoplexy. In terms of reducing the risk of disease, polyphenols regulate the flora, directly affect metabolism to promote the synthesis of SCFAs, and inhibit the production of TMA by anti-oxidation, anti-inflammation, and regulation of related metabolism, thereby reducing TMAO (31). It cleans free radicals with strong antioxidant capacity, reduces vascular endothelial damage, and maintains vascular health by inhibiting the production of inflammatory factors with anti-inflammatory properties. It can also regulate lipid metabolism, improve vascular endothelial function, and reduce the hidden dangers of stroke caused by atherosclerosis in many ways (207). In the rehabilitation stage of stroke patients, polyphenols can cross the BBB, reduce neuroinflammation, protect nerve cells, and promote the recovery of nerve function. At the same time, they can continuously improve vascular conditions, reduce the risk of thrombosis, regulate the immune and metabolic functions of the body to maintain the stability of the internal environment, and help patients recover better. It is a beneficial component to assist in the prevention and treatment of stroke, but it cannot replace regular medical treatment (208).

Omega-3 and omega-6 are essential polyunsaturated fatty acids for human body, which have a non-negligible effect on stroke (209). Omega-3 fatty acids (such as α-linolenic acid, EPA, DHA, etc.) can regulate blood lipids, reduce triglycerides, optimize the proportion of cholesterol, play an anti-inflammatory effect to reduce chronic inflammation, improve vascular endothelial function to ensure smooth blood flow, and reduce neuroinflammation, promote nerve cell regeneration, and improve hemorheology to help rehabilitation after stroke (210, 211). Omega-6 fatty acids (such as linoleic acid, etc.) are involved in normal physiological metabolism of human body and maintain cellular structure when they are present in an appropriate amount, but excessive intake can easily cause inflammatory response and increase the risk of stroke. In general, the reasonable intake ratio and amount of the two are of great significance for the prevention of stroke and the rehabilitation of patients (212). In addition, increasing the intake of food rich in omega-3 and omega-6 polyunsaturated fatty acids has shown positive effects. Fish, flaxseeds, and walnuts are rich in omega-3 and omega-6 polyunsaturated fatty acids, which have anti-inflammatory, anti-thrombotic, and neuroprotective effects. Supplementing omega-3 and omega-6 polyunsaturated fatty acids can improve cognitive and motor function recovery in stroke patients (213, 214).Takeo Sato et al. have also shown that omega-3 and omega-6 polyunsaturated fatty acids exhibit cardiovascular protective effects as well (215). Additionally, individualized nutritional support is also an important direction for dietary intervention. Tailored dietary plans are created for each patient, taking into account factors such as age, physical health, and any coexisting medical conditions, in order to provide the necessary nutrients for the body to recover effectively (216, 217).

Finally, we need to try to avoid diets high in salt, sugar, fat, excessive alcohol consumption, and diets low in dietary fiber, all of which may increase stroke risk. High-salt diet can increase the risk of stroke by increasing blood pressure, high-sugar diet can easily cause blood glucose metabolism disorders and accelerate vascular lesions, resulting in increased risk, high-fat diet can promote the formation of atherosclerotic plaques and induce the disease, excessive alcohol consumption can affect metabolism and stimulate blood vessels, and constipation and blood pressure fluctuations caused by lack of dietary fiber may also cause stroke (218, 219).

### 4.4 Drug therapy

In recent years, significant research advancements have been made in modulating the gut microbiota with pharmacological interventions to enhance stroke recovery. Initially, antibiotics are employed in specific instances to modulate the gut microbiota (220). While the administration of antibiotics necessitates careful consideration to prevent detrimental effects on microbiota balance, their judicious use in severely infected stroke patients can effectively eliminate pathogenic bacteria, thereby facilitating conditions conducive to subsequent gut microbiota reconstruction (221). For instance, rifaximin modulates the composition of the gut microbiota, enhancing the prevalence of *Ruminococcaceae* and *Lachnospiraceae*, elevating brain butyrate levels, and boosting the production of anti-inflammatory factors by microglia, thereby fostering neuroprotection (222). Secondly, increasing attention is being paid to developing small molecule compounds to regulate the metabolic activity or growth of specific gut bacteria. Gao et al. developed turmeric-derived nanovesicles (TNVs), and oral TNVs can exert anti-inflammatory effects by regulating the gut microbiota, repairing damaged intestinal barrier, and reshaping the phenotype of macrophages (223). Moreover, there has been a significant focus on utilizing traditional Chinese medicines(TCMs)to modulate the gut microbiota to facilitate stroke recovery. Many TCMs have the characteristics of multi-component and multi-target, and can comprehensively regulate the gut microbiota, immune system, and nervous system (224, 225). Certain TCMs formulations have been identified to augment the presence of beneficial microbiota, diminish the prevalence of pathogenic bacteria, and exhibit antioxidant, anti-inflammatory, and neuroprotective effects. For example, Ganluyin (GLY) has been shown to stabilize gut microbiota by increasing the levels of *Firmicutes* while decreasing the abundance of *Proteobacteria* and *Bacteroidetes*. Additionally, it enhances the integrity of the intestinal mucosal barrier in murine models and inhibits the LPS/TLR4/NF-κB inflammatory pathway originating in the gut (226). Another study found that Huanglian and Houpu have been shown to boost the levels of beneficial bacteria like *Akkermansia*, *Allobaculum*, *Alloprevotella*, and *Blautia*, while decreasing the levels of pathogenic bacteria such as *Shigella* and *Clostridium* spore-forming bacteria (227).

As technology advances and research deepens, adjusting the gut microbiota through pharmaceutical treatment may become an important component of the comprehensive treatment of stroke, bringing better prospects for recovery for patients.

## 5 Challenges and prospects

### 5.1 Challenges

Despite the swift advancements in MGBA research related to cerebral infarction, the field continues to encounter several challenges. These include a limited understanding of the underlying mechanisms, a paucity of clinical research, difficulties in developing effective treatment strategies, and constraints in detection technologies.

Initially, the intricate nature of signaling pathways is noteworthy. The signaling within the MGBA is facilitated through a multitude of pathways, encompassing the immune system, neurotransmitters, metabolic products, the enteric nervous system, and the vagus nerve, among others (38). Nevertheless, the current comprehension of the interactions among these pathways and their collective influence on the onset and progression of stroke remains insufficiently developed (228). For instance, while some studies have explored the effects of metabolic products such as SCFAs, BAs, and TMAO on the MGBA, the precise mechanisms of action and the interrelationships among these factors require further elucidation (41). Second, the impact of individual differences must be considered. The gut microbiota composition of each individual is distinct, influenced by a combination of genetic factors, dietary habits, lifestyle choices, and a multitude of other variables. This variability poses a significant challenge to the generalizability of research outcomes, complicating the development of standardized treatment protocols or preventive strategies (228, 229). Third, large-scale clinical trials are absent. Although certain studies have investigated the relationship between the MGBA and stroke, as well as the feasibility of related therapeutic approaches, no large-scale, multicenter clinical trials have been conducted to validate these findings (23, 230). This lack of robust clinical evidence hinders the widespread recognition and application of potential treatment strategies, such as FMT, whose safety and efficacy necessitate further clinical validation (92). Fourth, the uncertainty of probiotics and prebiotics. Probiotics and prebiotics are currently the focus of MGBA-based treatments, but their effects are uncertain. The role of different probiotic strains in stroke may be different, and the survival ability, colonization ability, and interaction with the host gut microbiota of probiotics need further research (231, 232). Fifth, the safety and ethical issues of FMT. FMT, as a relatively novel therapeutic approach, has shown potential in some studies but also has safety and ethical concerns (233). For example, the fecal matter transferred may contain pathogens, leading to infections and other complications; at the same time, strict standards and regulations are needed for the selection of fecal sources and donor screening (194). Sixth, the limitations of detection technologies. Currently, commonly used methods for gut microbiota detection include fecal sample analysis and gene sequencing, but these methods have certain limitations (234, 235). For example, fecal samples can only reflect part of the gut microbiota information and the collection, preservation, and processing of samples may affect the accuracy of detection results (236); although gene sequencing technology can provide more detailed microbial information, it is expensive and requires professional knowledge and skills for data analysis and interpretation (237, 238). Seventh, challenges in the detection of the MGBA function. The MGBA represents a complex detection methodology that explores the intricate interactions among gut microbiota, neurotransmitters, metabolites, intestinal barrier function, and immune indicators to investigate the relationship between the gut microbiota and the brain. Presently, there is a lack of direct and precise detection indicators and methodologies capable of accurately reflecting the interaction between the gut microbiota and the brain. This limitation hinders the investigation of the MGBA mechanism in cerebrovascular diseases (239, 240).

### 5.2 Prospects

The association between the MGBA and stroke is anticipated to be further clarified through integrative multiomics approaches, encompassing genomics, transcriptomics, proteomics, and metabolomics, among others, alongside the utilization of advanced neuroimaging modalities such as magnetic resonance imaging (MRI) and positron emission tomography (PET). The interplay between gut microbiota and the host, as well as the correlation between alterations in gut microbiota and modifications in brain structure and function, will be comprehensively examined to elucidate the underlying mechanisms (241, 242). In clinical practice, individualized treatment plans can be developed based on the composition and function of the patient’s gut microbiota, such as selecting probiotic strains or prebiotic interventions to address dysbiosis in the gut microbiota (243); combining MGBA-based therapy with traditional stroke treatment to achieve synergistic effects (178); developing drugs that target gut microbiota metabolites or directly regulate MGBA function; and improving prevention strategies by establishing early screening methods based on gut microbiota detection, intervening in high-risk populations promptly, and opening up new avenues for the prevention and treatment of stroke to improve patient outcomes (244).

## 6 Conclusion

In conclusion, the gut microbiota plays a vital role in the onset, progression, and management of cerebral infarction. Dysbiosis within the gut microbiota is significantly correlated with an increased risk of cerebral infarction, suggesting that modulation of the gut microbiota may provide innovative strategies for the prevention and treatment of this condition. Gut microbiota dysbiosis may elevate the risk of cerebral infarction through multiple mechanisms, including alterations in metabolic products, modulation of the immune system, and impacts on the neuroendocrine system. Conversely, cerebral infarction can exacerbate the imbalance of gut microbiota. Regarding prevention and therapeutic strategies, the utilization of probiotics and prebiotics, FMT, dietary interventions, and pharmacotherapy have demonstrated potential efficacy. These methodologies have enhanced the composition and functionality of the gut microbiota, thereby mitigating inflammation in patients and facilitating partial recovery of neural function. Nonetheless, these approaches remain contentious and present several issues requiring further investigation, particularly concerning the stability of therapeutic effects and safety. Future research should aim to elucidate the mechanisms underlying the interaction between the gut microbiota and cerebral infarction, thereby establishing a theoretical foundation for the development of more effective therapeutic interventions. This includes the creation of novel pharmacological agents that modulate the metabolic byproducts of the gut microbiota or regulate the signaling pathways between the gut microbiota and the brain. Additionally, it is essential to develop precise treatment plans tailored to the individual characteristics of patients. To facilitate the clinical application of gut microbiota regulation in the treatment of cerebral infarction, strengthening interdisciplinary collaboration is imperative. In summary, a complex relationship exists between the gut microbiota and cerebral infarction. Investigating the mechanisms underlying these interactions can advance our understanding of cerebral infarction pathogenesis and facilitate the development of innovative strategies for its prevention and treatment.
